# Antihyperglycemic activity of *Tectona grandis* Linn. bark extract on alloxan induced diabetes in rats

**DOI:** 10.4103/0974-7788.72488

**Published:** 2010

**Authors:** S. B. Varma, D. L. Jaybhaye

**Affiliations:** *Department of Pharmacology, Mahatma Gandhi Institute of Medical Sciences, Sewagram, Wardha - 402 102, India*; 1 *Department of Pharmacology, Mahatma Gandhi Mission Hospital, Aurangabad - 431 001, Maharashtra, India*

**Keywords:** Alloxan, anti-hyperglycemic activity, bark extract, diabetes, *Tectona grandis* Linn

## Abstract

*Tectona Grandis* Linn.(saag - tick wood), an indigenous medicinal plant, has a folk reputation among the Indian herbs as a hypoglycemic agent. The present study was carried out to evaluate the anti-hyperglycemic effect of *T. grandis* Linn. bark extract in control and alloxan-diabetic rats. Oral administration of the bark suspension of *T. grandis* (2.5 and 5 g/kg body wt.) for 30 days resulted in a significant reduction in blood glucose (from 250 ± 6.5 to 50 ± 2.5 mg/dL). Thus, the present study clearly shows that the *T. grandis* Linn. bark extract exerts anti-hyperglycemic activity.

## INTRODUCTION

Diabetes mellitus is the most common disease associated with deranged carbohydrate metabolism, affecting about 200 million people worldwide.[1] Extracts of various plant materials with a potential of decreasing the blood sugar have been tested in experimental animal models and their effects confirmed.[2] Many unknown and lesser known plants are used in folk and tribal medicinal practices in India. The medicinal values of these plants are not much known to the scientific world. *Tectona grandis* (saag – tick wood),(family Verbenaceae) is one such medicinal plant.

According to Ayurveda, the wood of *T. grandis* is acrid, cooling, laxative, sedative to gravid uterus and is useful in the treatment of piles, leukoderma and dysentery. Roots are useful in anuria and retention of urine.[3,4] The flowers are acrid, bitter, dry and cure bronchitis, biliousness, urinary discharges, etc.[3] According to Unani system of medicine, its oil is useful in scabies, whereas the wood is best used for headache, biliousness, burning sensation and pain and liver-related troubles.[3] It allays thirst, and acts as an anthelmintic, expectorant and anti-inflammatory agent.[3,4] The bark is astringent, acrid, cooling, constipating, anthelmintic and depurative. It is useful in bronchitis, hyperacidity, vitiated conditions of pitta; dysentery, verminosis, burning sensation, diabetes, leprosy and skin diseases.[5]

*T. grandis* is known as saag in Sanskrit, sagun in Hindi, sagwan in Marathi and teak tree in English.[4] Lapachol, a naphthaquinone isolated from the roots of *T. grandis*, has an anti-ulcerogenic effect on experimental gastric and duodenal ulcers induced subsequently in rats and guinea-pigs.[6] *T. grandis* sawdust extract inhibits the growth of *Aspergillus niger*; the active compounds were identified as deoxylapachol and tectoquinnone.[7] *T. grandis* has been investigated for its nitric oxide scavenging activity[8] and wound healing activity in rats.[9] *T. grandis*

contains tannin, which is used as an anti-inflammatory agent and is also used topically for the treatment of burns.[10,11]

### Phytochemistry study

*T. grandis* wood contains, in its cavities, white crystalline deposits of calcium phosphate, silica and ammonium and magnesium phosphates, which are also resins. Seed contains a bland fatty oil.[4] Lapachol is a naphthoquinone and lapachonone, found in *Tectona* wood and bark,[6,11] has anti-hyperglycemic effect.[12] *T. grandis* Linn. sawdust contains deoxylapachol and tectoquinnone as active components.[7,13,14]

In the light of the above information, the present investigation was undertaken to evaluate the anti-hyperglycemic potential of *T. grandis* Linn. bark extract on fasting blood sugar levels in alloxan-induced diabetic rats

## MATERIAL AND METHODS

### Plant material and preparation of extract

The fresh stems of the plant *T. grandis* were collected from the campus of Mahatma Gandhi Institute of Medical Sciences, Wardha (MS) India, and confirmed by a local botanist. The stems were shade dried and made into a fine powder. The powder was macerated for 24 hours in 70% v/v ethanol. Then they were subjected to percolation by using 70% v/v ethanol as a solvent. Percolated solution was again shade dried and the extract was used to prepare an aqueous solution in the desired concentration just before use every time

### Experimental animals

Male albino wistar rats (150–200 g) and albino mice (25–35 g) were used. Animals were procured from Pharmacy college of Borgaon (Meghe) Wardha National Center after taking permission from animal ethical committee of

Mahatma Gandhi Institute of Medical Sciences. The animals were fed on a pelleted diet and water, and the temperature of the animal room was maintained at 37C. All these facilities were provided by Department of Pharmacology, Mahatma Gandhi Medical Sciences. The animals were maintained in their respective groups. All the studies were conducted in accordance with the National Institute of Health’s Guide for the Care and Use of Laboratory Animals.[15]

### Toxicity studies

Acute toxicity study was conducted in equal number of male albino mice (n=6) using the doses of 150, 300, 600, 1200, 2400, 4800, 5500 mg/kg *T. grandis* bark extract according to OECD guidelines. The animals were observed for 72 hours to note any changes in behavioral pattern including the level of consciousness, gait, food and water intake and mortality. Adverse effects were not observed in the above doses.

During our study period, there were no reports available on the *T. grandis* bark extract. For this reason, we have done acute toxicity study of *T. grandis* Linn. bark extract.

### Collection of blood sample

Collection of blood sample was done in the rats by tail venipuncture. In restrained (unanesthetized) animals, lateral or dorsal veins are dilated by dipping the tail into water at 40 to 50°C or by rubbing with xylol and then cleaning the part with spirit. The tail was grasped between the thumb and index finger, and a needle (25 to 27-gauge and 0.5–1 inch long fitted with 1 or 2 mL syringe) is introduced near the distal portion of tail with bevel up. Gentle aspiration was applied to avoid collapse of wall of vein obliterating the needle opening. After collecting the blood sample, local antibiotic was applied over the wound along with a sterile bandage for avoiding infection of wound. Blood sample from retro orbital plexus was collected when the blood sample from tail was not sufficient or when the tail vein was totally damage.[16]

Blood glucose levels were estimated using an electronic glucometer (prestige) manufactured by American Diabetic Supply._The sensitivity of electronic glucometer is 0.5 mg/dL as compared to laboratory reading.

### Experimental induction of diabetes

Diabetes was induced in rats by a single intra-peritoneal injection of alloxan monohydrate (150 mg/kg). Alloxan was first weighed individually for each animal, according to its weight, and solubilized with 0.2 mL saline (154 mM NaCl) just prior to injection. Twenty four hours after alloxan injection,[17] rats with diabetes hyperglycemia with a blood glucose range of 250–300 mg/dL were used for this experiment. All the animals (albino wistar rats) used in our study developed diabetes mellitus after alloxan injection.

Treatment with plant extract was started 24 hours after alloxan injection. Blood samples were drawn at 48 hours, 15 days and 30 days till the end of the study (30 days).

### Experimental design

Animals were divided into six groups of six rats in each. Food and water were provided *ad libitum* to the animals. The grouping is as follows.

- Group 1: Control vehicle: 2% gum acacia
- Group 2: Diabetic control
- Group 3: Control + *T. grandis* suspension (2.5 g/kg body wt.)
- Group 4: Diabetic + *T. grandis* suspension (2.5 g/kg body wt.)
- Group 5: Diabetic + *T. grandis* suspension (5 g/kg body wt.)
- Group 6: Diabetic + glibenclamide (10 mg/kg per day).

Treatment with plant extracts was started 24 hours after alloxan injection. After 48 hours, 15 and 30 days of treatment, the rats were fasted overnight and blood glucose was tested in morning.

### Statistical analysis

All the values of fasting blood sugar were expressed as mean ± standard error mean (SEM) and analyzed using one way ANOVA.

## RESULTS

Changes in blood glucose levels on treatment of diabetic rats with *T. grandis* bark extract and glibenclamide are presented in Table 1. A significant increase in blood glucose is observed in diabetic rats when compared with control rats. Oral administration of *T. grandis* (2.5 and 5 g/kg body wt.) for 30 days shows a significant reduction in blood glucose in diabetic rats, when compared with untreated diabetic rats.

**Table 1:** Changes in blood glucose in control and alloxan diabetic rats treated with *T. grandis* bark extract and glibenclamide

Groups	Blood glucose (mg/dL) (mean ± SEM)		
	48 hours	15 days	30 days
Control (2% gum acacia)	45 ± 3.7	45 ± 3.7	60 ± 3
Diabetic control	282 ± 9	280 ± 8	250 ± 6.5
Control+ TG (2.5 g/kg body wt.)	45 ± 2.2	45 ± 3.5	45 ± 3.7
Diabetic + TG (2.5 g/kg body wt.)	280 ± 9	180 ± 4	68 ± 3**
Diabetic + TG (5 g/kg body wt.)	278 ± 4	100 ± 4	50 ± 2.5***
Diabetic + glibenclamide (10 mg/kg per day)	250 ± 4.5	100 ± 3.5	40 ± 2***

## DISCUSSION

Blood sugar level was increased as expected in alloxan-injected animals, since alloxan causes a massive reduction in insulin release by the destruction of the β-cells of the islets of Langerhans and inducing hyperglycemia.[18] Oral administration of *T. grandis* bark extract (2.5 and 5 g/kg body wt.) resulted in a significant reduction in the blood glucose levels.

The number of functionally intact β-cells in the islet organ is of decisive importance to the development course and outcome of diabetes. The renewal of β-cells in diabetes has been studied in several animal models.[19] The total β-cell mass reflects the balance between the renewal and loss of these cells. It was also suggested that regeneration of islet β-cells following destruction by alloxan may be the primary cause of the recovery of alloxan-injected guinea pigs from the effects of the drug.[20] In alloxan-induced diabetes, (–)-epicatechin[21] and *Vinca rosea* extract[22] have also shown to act by β-cell regeneration. Similar effects in streptozotacin-treated diabetic animals have been reported on treatment with pancreas tonic,[23] ephedrine,[24] and *Gymnema sylvestre* leaf extracts.[19] Some studies have also reported the regeneration of islets’ β-cells by glibenclamide.[25]

The anti-hyperglycemic activity of *T. grandis* bark extract may be due to the regeneration of islets’ β-cells following destruction by alloxan, as the extract shows significant reduction of blood glucose levels in 15 and 30 days, at a dosage of 2.5 and 5 g/kg body wt., an effect similar to that of glibenclamide. But the *T. grandis* bark extract was more effective at a dose of 5 g/kg body wt. Glibenclamide is standard drug causes decrease in blood glucose 40 ± 2 mg/dl on 30^th^ day while the *T. grandis* bark extract in the doses of 2.5gm/kg body wt. and 5gm/kg body wt. also decrease the blood glucose 68 ± 3 and 50 ± 2.5 respectively on 30^th^ day. This antihyperglycemic effect may be due to lapachol (a naphthoquinone), lapachonone,[6,12] deoxylapachol and tectoquinnone,[7] which have been reported to be the constituents of *T. grandis*.[12]

In conclusion, we have demonstrated that the neglected medicinal plant *T. grandis* possesses anti-hyperglycemic effects. Further active research is underway in our laboratory to elucidate the mechanisms of action of this medicinally important plant.
